# CD73^+^ Mesenchymal Stem Cells Ameliorate Myocardial Infarction by Promoting Angiogenesis

**DOI:** 10.3389/fcell.2021.637239

**Published:** 2021-05-12

**Authors:** Qiong Li, Huifang Hou, Meng Li, Xia Yu, Hongbo Zuo, Jianhui Gao, Min Zhang, Zongjin Li, Zhikun Guo

**Affiliations:** ^1^Henan Key Laboratory of Medical Tissue Regeneration, Xinxiang Medical University, Xinxiang, China; ^2^Xinxiang Central Hospital, Xinxiang, China; ^3^Department of Hepatobiliary Surgery, Affiliated of Cancer Hospital of Zhengzhou University, Zhengzhou, China; ^4^Nankai University School of Medicine, Tianjin, China

**Keywords:** CD73, VEGF, adipose derived mesenchymal stem cells, angiogenesis, myocardial infarction

## Abstract

With multipotent differentiation potential and paracrine capacity, mesenchymal stem cells (MSCs) have been widely applied in clinical practice for the treatment of ischemic heart disease. MSCs are a heterogeneous population and the specific population of MSCs may exhibit a selective ability for tissue repair. The aim of our research was to adapt the CD73^+^ subgroup of adipose derived MSCs (AD-MSCs) for the therapy of myocardial infarction (MI). In this research, AD-MSCs were isolated from adipose tissue surrounding the groin of mice and CD73^+^ AD-MSCs were sorted using flow cytometry. To investigate the therapeutic effects of CD73^+^ AD-MSCs, 1.2 × 10^6^ CD73^+^ AD-MSCs were transplanted into rat model of MI, and CD73^–^ AD-MSCs, normal AD-MSCs transplantation served as control. Our results revealed that CD73^+^ AD-MSCs played a more effective role in the acceleration function of cardiac recovery by promoting angiogenesis in a rat model of MI compared with mixed AD-MSCs and CD73^–^ AD-MSCs. Moreover, with the expression of CD73 in AD-MSCs, the secretion of VEGF, SDF-1α, and HGF factors could be promoted. It also shows differences between CD73^+^ and CD73^–^ AD-MSCs when the transcription profiles of these two subgroups were compared, especially in VEGF pathway. These findings raise an attractive outlook on CD73^+^ AD-MSCs as a dominant subgroup for treating MI-induced myocardial injury. CD73, a surface marker, can be used as a MSCs cell quality control for the recovery of MI by accelerating angiogenesis.

## Background

Various clinical trials have been conducted on patients with acute myocardial infarction (MI) using multiple cell lines, including mesenchymal stem cells (MSCs), bone marrow stem cells, and cardiac resident stem cells (Mayfield et al., 2014; Tao et al., 2016; Miao et al., 2017). These therapies utilizing stem cells to regenerate lost cardiac muscle are clinically attractive. The use of MSCs, multipotent differentiation potential stem cells derived from mesoderm, for cell therapy might play a cardioprotective effect. This effect was not through the production of new cardiac tissue, but through the secretion of secondary paracrine factors that enhance the endogenous repair pathways (Bobi et al., 2017; Naji et al., 2019). Clinical and preclinical trials’ results showed that the transplanting effect of MSCs was very limited due to its poor cell persistence and cardiomyocyte differentiation (Chen et al., 2018; Li et al., 2020).

Mesenchymal stem cells are heterogeneous and could be divided into many subgroups with different biological characteristics (Du et al., 2016; Mo et al., 2016; Chin et al., 2018; Liu et al., 2019). Morphologically, MSCs could be divided into three subgroups: a small fast self-renewing cell subgroup, a fibroblast-like subgroup, and a large slow-growing square or flat cell subgroup (Haasters et al., 2009). Functionally, CD56^+^ MSCs were proliferative, more chondrogenic, and less adipogenic than CD56^–^ MSCs (Elahi et al., 2016). Tseng reported that CD105^+^-AD-MSCs were inclined to differentiate into chondrocytes (Tseng et al., 2020). At present, bulk MSCs were used in most *in vivo* and *in vitro* studies, and the heterogeneity of unsorted MSCs compromised to comparability of cell therapy studies (Dromard et al., 2014; Fu et al., 2019).

Mesenchymal stem cells exhibit a variety of immunophenotypes. CD44, CD73, CD29, CD90, and CD105 are the most common markers of MSCs, which also lack the cell surface antigen CD45 (Petrenko et al., 2020). As reported by Florian et al., the expression patterns of CD73, CD105, CD90 varied in different morphological subsets. All three markers were stably expressed in rapid self-renewal cells whereas most flat cells lacked one or more MSC surface markers, predominantly CD73 (Haasters et al., 2009). In addition, there were significant differences in CD73 expression in MSCs from different sources (Darzi et al., 2016). We have found that CD73^+^ MSCs were the dominant subgroup regarding the potential for myocardial differentiation (Li et al., 2013).

CD73, an ecto-5′-nucleotidase, is a glycoprotein anchored to the cell membrane by glycosyl-phosphoryl inositol. It was widely expressed on the cell membrane and implicated in signal transduction (Allard et al., 2017; Kordass et al., 2018). Previous researches have proved that CD73 had the potency to promote cell proliferation and that CD45^–^CD14^–^CD73^+^cell subsets had stronger clone forming ability (Suto et al., 2017, 2020). CD73 could promote tumor angiogenesis, release cytokines under ischemic conditions, and has the ability to make tumor cells escape immune recognition (Beavis et al., 2012; Ghalamfarsa et al., 2019). CD73 played an important role in myocardial protection during myocardial ischemic preconditioning (Wolff et al., 2015), and the expression of CD73 was upregulated in immune cells after MI and was linked to myocardial repair and improvement of cardiac function (Tan et al., 2019). In conclusion, CD73 may be a sensitive marker for screening subsets of MSCs and treating myocardial injury.

Adipose derived mesenchymal stem cells had many similarities with MSCs from alternative sources (Zuk et al., 2001). Among all transplantable seed cells, AD-MSCs had the peculiarities of simple sampling, multi-directional differentiation, few ethical problems, rapid proliferation *in vitro*, and low immunogenicity (Bobi et al., 2017). Therefore, AD-MSCs have become a main choice for stem cell therapy of MI. Most studies have utilized unsorted AD-MSCs for research and injury treatment. Although CD73 has been used as an important criterion for the identification of AD-MSCs for many years, its specific role in the treatment of AD-MSCs transplantation is still unclear (Calloni et al., 2013; Ode et al., 2013). The role of CD73 in AD-MSCs cardiomyocyte differentiation and cytokine secretion also needs to be clarified.

In this study, CD73^+^ and CD73^–^ subsets of AD-MSCs were sorted by flow cytometry and their morphology and genomics were evaluated in detail. Lentivirus and APCP (inhibitor of CD73) were used to alter the expression or activity of CD73 and then the changes in cytokines secretion of these AD-MSCs were observed *in vitro*. We also evaluated the effect of CD73^+^AD-MSCs transplantation on the repair of myocardial infarction in rats. The main objective of this study was to evaluate the utility of the dominant subset of AD-MSCs for improving MI treatment, and to provide theoretical and experimental basis for clinical application of CD73^+^ cells.

## Materials and Methods

### Culture, Sorting, and Detection of AD-MSCs, CD73^+^, and CD73^–^ AD-MSCs

Adipose derived mesenchymal stem cells were isolated and cultured from the groin adipose tissue of adult C57BL/6 mice (body weight: 28.20 ± 0.66 g), which were obtained from the Experimental Animal Center of Xinxiang Medical University. The upon experimental methods and reagents used were referred to the previously published protocol (Zuk et al., 2001; Li et al., 2013). Cells were cultured with low sugar DMEM (Dulbecco’s modified Eagle’s medium) containing 10% FBS (Fetal bovine serum; Hyclone, United States). When cells reached 80% confluence, they were passaged using 1:2 splits. The morphology of the cells was observed by inverted microscope. Immunofluorescence and flow cytometry analysis were conducted on passage 3 AD-MSCs using monoclonal antibodies against CD29, CD44, CD73, and CD45 (BD Bioscience, 1:500).

CD73^+^AD-MSCs and CD73^–^ AD-MSCs were isolated using flow cytometry and then cultured. With CD73 as the standard, flow cytometry (FACS Aria II, BD Bioscience) was used to sort out the two subgroups. The morphology of CD73^+^ AD-MSCs and CD73^–^AD-MSCs in primary culture was determined by using HE staining. Subpopulations were further subcultured and the cells from the fourth to sixth passages were used for the follow-up experiments. Quantitative real-time PCR and Western blotting were used to detect the expression of CD73 in the two subgroups.

### Real-Time PCR

Total RNA from CD73^+^ AD-MSCs and CD73^–^AD-MSCs were extracted with Trizol (Invitrogen Corporation, China) according to the manufacturer’s instructions. First-strand cDNA was synthesized with 50 ng of RNA (Takara Biomedical Technology, China), which was used for each real-time PCR reaction. Relative quantitation of mRNA by real-time PCR was performed using Applied BeyoFast^TM^ SYBR Green qPCR Mix (2X) (Beyotime Biotechnology, China). The primer sequences were as follows. CD73, 269bp, Forward primer: GGTTGTGGGGATTGTTGGATA, Reverse primer: GCACTTCTTTGGAAGGTGGAT.

GAPDH, 231bp, Forward primer: TGGTGAAGGTC GGTGTGAAC, Reverse primer: GCTCCTGGAAGATGGT GATGG.

### Western Blot Analysis

CD73^+^ AD-MSCs and CD73^–^ AD-MSCs were lysed in RIPA lysis buffer. Total protein was extracted and subjected to electrophoresis using 10% SDS-polyacrylamide gels and transferred to an apolyvinylidene difluoride (PVDF) membrane. The membrane was incubated with primary antibodies overnight and then incubated with secondary antibody for 2 h at room temperature. The primary antibodies included CD73 (ab175396, Abcam Biotechnology, United Kingdom, 1:500) and β-actin (AF0003, Beyotime Biotechnology, China, 1:1000). ECL Plus kit (P0018S; Beyotime Biotechnology, China) was used to detect signals according to the manufacturer’s instructions.

### Microarray Analysis of CD73^+^ and CD73^–^ AD-MSCs

The fifth passages of sorted subpopulations were used for microarray analysis. Gene chip was used to detect differentially expressed genes with consequent gene ontology (GO) analysis (Jikai Biotechnology Co., China). Genes in the GO term (including cellular components, biological processes and molecular functions) with *P* < 0.05 were considered significantly enriched. The Kyoto Encyclopedia of Genes and Genomes (KEGG) is a database resource that clarifies the effects and advanced functions of biological systems. This resource was used to identify significant differences in differentially expressed genes in KEGG pathways. The datasets presented in this study can be found in online repositories: NCBI GEO; GSE167219

### Overexpression and Inhibition the Expression of CD73

CD73 gene overexpression and CD73 gene siRNA lentivirus were constructed by Jikai Biotechnology Co., The cells in logarithmic growth phase (amount 5 × 10^4^) were seeded in a 6-well cell culturing plate in complete culture medium in incubator supplied with 5% CO_2_ at 37°C. Upon achieving 30–40% confluence, appropriate amount of medium virus enhanced infection solution (ENi.S.) and polybrene were added according to the MOI (Multiple of infection) value of cells. After 72–96 h infection time, lentivirus reporter gene GFP expression were detected. The Lentiviral transfected cells were used for subsequent VEGF detection by ELISA.

### Paracrine Effects of CD73^+^ AD-MSCs

All experimental groups (AD-MSCs, CD73^–^AD-MSCs, CD73^+^AD-MSCs and CD73^+^ AD-MSCs + APCP) were cultured in complete medium. Upon reaching 80–90% confluence, cells were cultured in serum-free DMEM. APCP (80 μmol/L; alpha, beta—methyleneadenosine-5′- diphosphate) was used to inhibit the activity of CD73 in CD73^+^AD-MSCs + APCP group. After 24 h, the concentration of cytokines in the conditioned medium was measured by ELISA. The selecting principles for cytokines were as follows: the cytokines which were secreted by MSCs and closely related to myocardial repair including vascular endothelial growth factor (VEGF), stromal cell-derived factor-1α (SDF-1α), basic fibroblast growth factor (bFGF), and hepatocyte growth factor (HGF) (Ho et al., 2016; Naji et al., 2019). Cytokine detection kit was purchased from Wuhan Huamei biotech Co., Ltd.

### Scratch Wound Healing Assay

To test the ability of CD73^+^AD-MSCs to induce endothelial migration, HUVEC cell line was used for scratch wound healing assay. 10^6^ cells HUVECs were seeded on a 6-well plate in Endothelial Cell Medium (ScienCell, United States) supplemented with 5% FBS. The cells were left 24 h to reach 80% confluence. The monolayer was disrupted by using a sterile 100 ul tip and an acellular line was left in the middle of the well. Then HUVECs were treated with the conditioned medium of CD73^+^AD-MSCs and CD73^–^AD-MSCs. Images for five fields of views for each scratch were taken at 0 and 6 h. The percent of wound closure was calculated using Image J.

### Myocardial Infarction Model and Cell Transplantation

Adult mix gender SD rats (body weight: 230 ± 18 g) were obtained from the Experimental Animal Center of Xinxiang Medical University and then used to replicate the MI model. The MI model was replicated as follows. After transnasal anesthesia with aether, the rats were immobilized in the supine position and anesthetized by intraperitoneal injection with 1% pentobarbital sodium (40 mg/kg). After skin preparation and disinfection, a median tracheotomy and intubation were performed. Make an incision between the third or fourth rib on the left to open the chest and expose the heart. At the lower 1–2 mm of the junction between the left atrial ear and the pulmonary artery cone, the left anterior descending coronary artery (LAD) was ligated by a 5/0 silk suture. Then the thorax was closed and the rats were injected 1.6 million units of penicillin into the abdominal cavity. After resumption of spontaneous breathing, the animal was monitored and tracheal intubation was removed. AD-MSCs were trypsinized, centrifuged, and resuspended before transplantation. 1.2 × 10^6^ cells were injected into myocardial tissue of the infarcted areas in each group. The rats were randomly divided into sham operation group (SHAM), MI, mixed AD-MSCs transplantation group (MI + AD-MSCs), CD73^+^ AD-MSCs transplantation group (MI + CD73^+^ AD-MSCs) and CD73^–^AD-MSCs transplantation group (MI + CD73^–^AD-MSCs). Each group had 6 rats. All experiments complied with the Ethics Committee of Xinxiang Medical University (XYLL-2015023).

### Color Doppler Ultrasound Detection of MI Postoperative Cardiac Function in Rats

PHILPS iE33 ultrasound apparatus and S12-4 heart probe were used to evaluate the cardiac function every week from 1st to 4th week after MI. 1% pentobarbital sodium (40 mg/kg) was injected intraperitoneally before the analysis. The evaluated indicators of ultrasonic diagnosis were as follows: left ventricular ejection fraction (LVEF), left ventricular end-systolic volume (LVESV), left ventricular end-diastolic volume (LVEDV).

### The Expression of VEGF and Factor VIII After MI *in vivo*

The myocardial tissue including infarcted area, infarcted marginal and peri-infarcted area were harvested, fixed in 4% buffered formalin, embedded in paraffin and then sliced continuously with the thickness of 8 μm. VEGF and factor VIII were detected by immunohistochemistry staining and Western blotting. The primary antibodies were monoclonal antibody against VEGF (bs-1313R, Beijing BOOSEN Biotechnology Co., Ltd., China, 1:200) and polyclonal antibody against VIII factor (bs-2974R, Beijing BOOSEN Biotechnology Co. Ltd., China, 1:200). In brief, the sections were deparaffined, rehydrated, quenched with 3% H2O2, and blocked with 10% normal serum, then incubated with above primary antibodies overnight at 4°C, followed by incubation with secondary antibody and SABC-HRP Kit (Beyotime Biotechnology, China). The staining was developed by using 3,3N-diaminobenzidine Tertrahydrochloride and finalized with lightly counter staining with hematoxylin. 10 visual fields were randomly selected from each slide and analyzed by motic Images advanced software. Immunocytochemical staining positive products were quantitatively analyzed by HMIAS-2000 high definition medical image and text analysis system. The intensity of the positive reaction product was analyzed by the integral optical density (IOD) of the image processing system. Western blotting was used to detect VEGF and factor VIII in 120 mg myocardium from the edge of the left ventricular infarction at the end of the 4th week after MI.

### Data Analysis

SPSS 23.0 statistical software were used for statistical analyses. The data were presented as means ± standard deviation (SD). The difference between groups was analyzed using one-way ANOVA and Tukey’s *t*-test. *P* < 0.05 was considered to be statistically significant.

## Results

### Morphological Characteristics and Immunophenotyping of AD-MSCs

**FIGURE 1:**
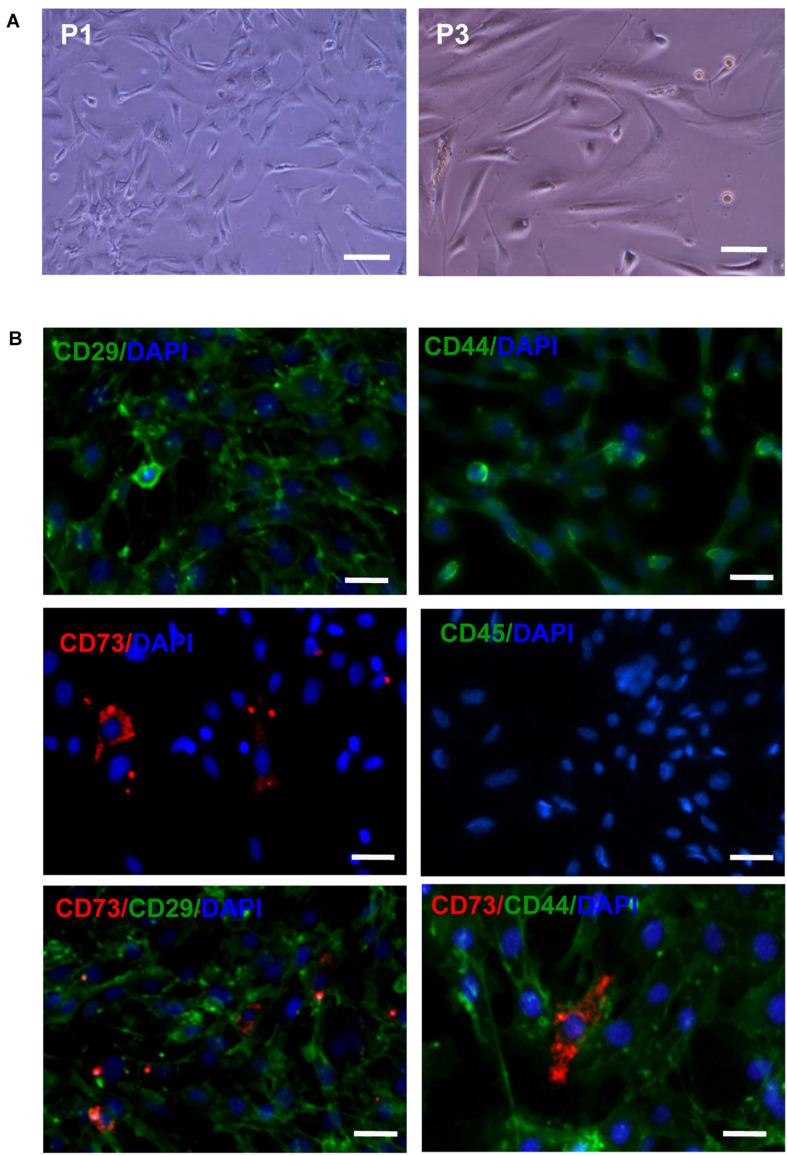
The morphology and phenotypical characterization of AD-MSCs. **(A)** Morphology of AD-MSCs at different passages. P1: passage 1, P3: passage 3; Scale bar, 20 μm. **(B)** Phenotypical characterizations of AD-MSCs using multicolor immunofluorescence. Immunofluorescence was performed for surface markers CD29, CD44, CD73, and CD45. Nuclei were counterstained with DAPI. Scale bars, 10 μm.

After the primary cells were cultured for 30 min, a large number of suspended circular cells were visible under the inverted microscope. After 2–3 days, the growth medium was replaced for the first time and AD-MSCs were assumed short rod or round shapes. After 7–10 days, AD-MSCs reached up to 80% confluence. At passage 3 to 6 the cells were mostly spindle shaped and polygonal. The majority of passage 3 AD-MSCs expressed CD29 and CD44 (81.3 and 99.1%, respectively), while only 15.7% of cells expressed CD73 and CD45 was virtually undetectable (Figure 1 and Supplementary Figure 1).

### Characterization of CD73^+^ and CD73^–^ Subgroups

**FIGURE 2:**
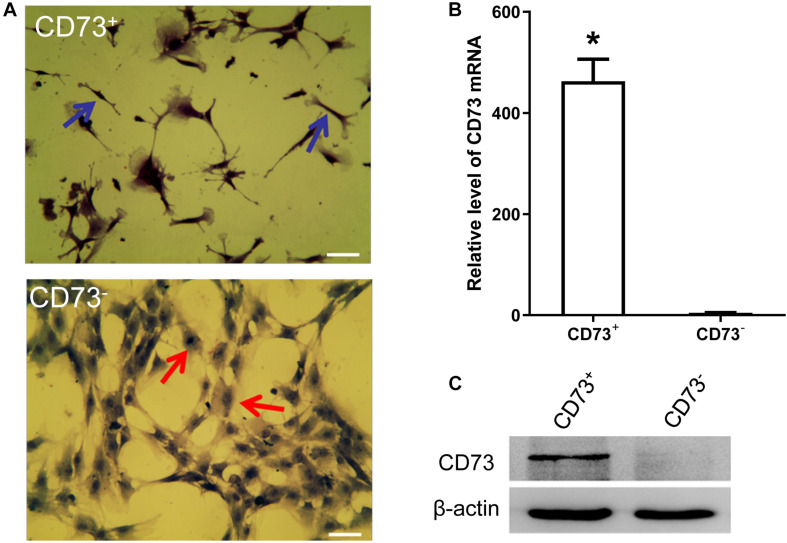
AD-MSCs can be divided into CD73^+^ and CD73^–^ subpopulations. **(A)** HE staining was used to detect the morphological changes of CD73 ^+^ and CD73^–^ AD-MSCs. Blue arrows indicated spindle cells, red arrows showed big polygonal cells. Scale bars, 20 μm. **(B)** Detection of CD73 mRNA in CD73^+^ and CD73^–^ AD-MSCs by Real-time quantitative PCR. Results are presented as mean ± SD.*P < 0.05, vs. CD73^–^ AD-MSCs. **(C)** The expression of CD73 in CD73^+^ and CD73^–^ AD-MSCs by Western blotting.

**FIGURE 3:**
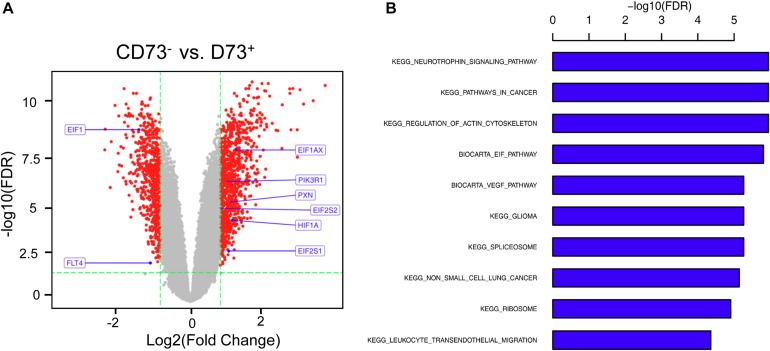
Differential gene analysis of CD73^+^ vs. CD73^–^AD-MSCs. **(A)** Volcano map of differential genes. **(B)** Go analysis for differentially expressed genes by KEGG.

CD73^+^AD-MSCs exhibited mainly spindle and rod-like shapes, whereas CD73^–^AD-MSCs mainly polygonal large cells (Figure 2A). The expression of CD73 in CD73^+^AD-MSCs and CD73^–^AD-MSCs has statistically significant differences (*P* < 0.05) (Figures 2B,C). These subtypes also displayed significant differences in gene expression patterns. Based on GO analysis, the differences concerned 10 signaling pathways, including VEGF pathway which were associated with pro-angiogenesis of AD-MSCs (Figure 3).

### Effect of CD73 on Cytokine Secretion of AD-MSCs *in vitro*

**FIGURE 4:**
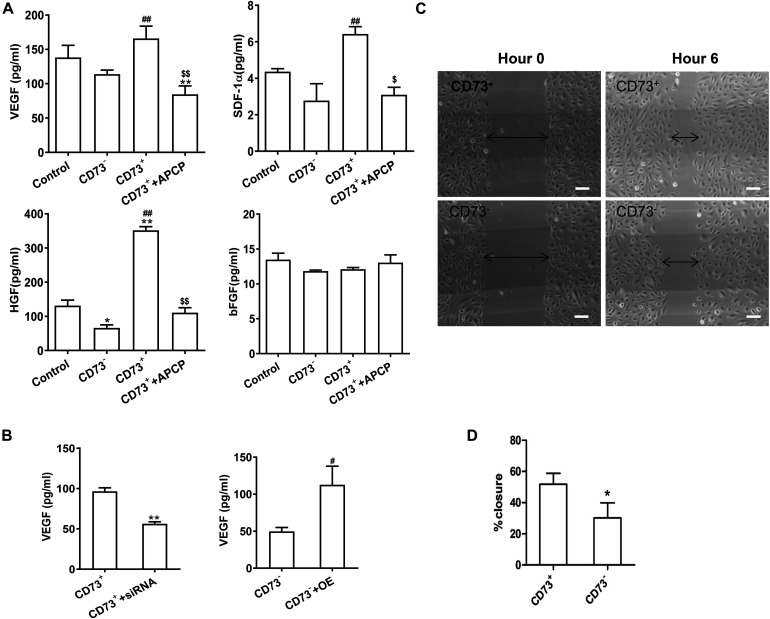
Pro-angiogenetic effects of CD73^+^ AD-MSCs. **(A)** Cytokine secretion analysis by ELISA in unsorted AD-MSCs (control), CD73^+^AD-MSCs, CD73^–^ AD-MSCs, and CD73^+^AD-MSCs + APCP. *P < 0.05, **P < 0.01, vs. control; ^#^*P* < 0.05, ^##^*P* < 0.01, vs. CD73^–^ AD-MSCs; ^$^*P* < 0.05, ^$$^*P* < 0.01, vs. CD73^+^ AD-MSCs; **(B)** VEGF secretion of cytokines of AD-MSCs with changing CD73 expression. CD73^+^ + siRNA: CD73^+^ AD-MSCs were transfected with CD73 gene SiRNA lentivirus; CD73^–^ + OE: CD73^–^ AD-MSCs were transfected with CD73 gene overexpression lentivirus. ** *P* < 0.01, vs. CD73^+^AD-MSCs; ^#^*P* < 0.05, vs. CD73^–^ AD-MSCs. **(C)** Scratch wound healing assay of HUVEC treated with conditioned media of CD73^+^ AD-MSCs and CD73^–^ AD-MSCs. **(D)** The percent of wound closure was calculated. *P < 0.05 vs. CD73^–^ AD-MSCs. Scale bars, 100 μm.

The secretion of VEGF, SDF-1α, and HGF in CD73^+^AD-MSCs was significantly higher than that of CD73^–^AD-MSCs(*P* < 0.01) while secretion of bFGF remained similar between these two subgroups (*P* > 0.05). Inhibition of CD73 activity of CD73^+^AD-MSCs by APCP resulted in decreased secretion of VEGF (*P* < 0.01), SDF-1α (*P* < 0.05), HGF (*P* < 0.01) and no effect on that of bFGF (*P* > 0.05). The expression of SDF-1α and HGF in CD73^–^AD-MSCs was lower than that in unsorted AD-MSCs (Figure 4A). When CD73 expression was down-regulated in CD73 positive subset, VEGF secretion was decreased (*P* < 0.01) while it was vice versa in CD73 negative subset (*P* < 0.05) (Figure 4B). Scratch wound healing assay was used to evaluate the ability of cellular products to support angiogenesis *in vitro*. When HUVEC was treated with CD73^+^AD-MSCs and CD73^–^AD-MSCs conditioned medium for 6 h, the percent of wound closure was 51.8 and 30.2%, respectively. The difference between CD73^+^AD-MSC and CD73^–^AD-MSC induced wound closure was statistically significant (Figures 4C,D). These results indicate that CD73 promotes secretion of VEGF, SDF-1α, and HGF factors.

### Results of Cardiac Function Test

**FIGURE 5:**
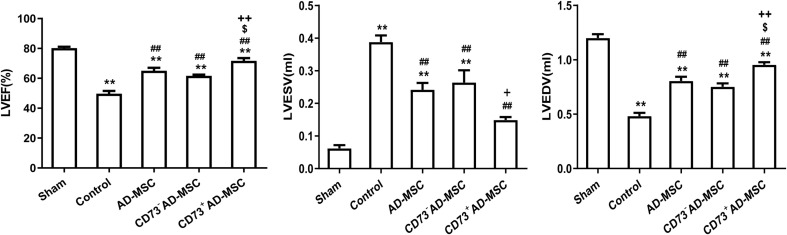
Improvement of cardiac function after CD73^+^AD-MSCs, CD73^–^ AD-MSCs, and AD-MSCs transplantation at week 4. Cardiac hemodynamic monitoring. LVEF: Left ventricular ejection fraction; LVESV (ml): left ventricular end-systolic volume; LVEDV (ml): left ventricular end-diastolic volume. *P < 0.05, **P < 0.01, vs. Sham; ^##^*P* < 0.01, vs. Control; ^[*dollar*]^*P* < 0.05, vs. AD-MSCs; ^+^*P* < 0.05, ^++^*P* < 0.01, vs. CD73^–^ AD-MSCs, *n* = 5∼6. One-way ANOVA, then followed by post Tukey’s test for multiple comparisons. Data are presented as mean ± SD.

Compared with the sham group, the LVEF and LVEDV significantly decreased (*P* < 0.01) and the LVESV significantly increased at 1–4 weeks after MI (*P* < 0.01). Compared with MI group, however, the LVEF and LVEDV in MI rats from cell transplantation group were significantly increased (*P* < 0.01). Importantly, the LVEDV did not significantly increase at the 1st and 2nd weeks post-MI in CD73^–^ cells transplantation group (*P* > 0.05). The LVESV significantly decreased at the 2nd and 4th weeks in all three cell transplantation groups (*P* < 0.01). Compared to AD-MSCs and CD73^–^AD-MSCs groups, CD73^+^AD-MSCs transplanted rats exhibited increase of LVEF (1st, 2nd, and 4th weeks) and LVEDV (4th week) (*P* < 0.05). When compared with AD-MSCs group, the LVESV of CD73^+^AD-MSCs group decreased on 2nd weekend (*P* < 0.05). These results indicated that CD73^+^AD-MSCs, AD-MSCs and CD73^–^AD-MSCs could significantly improve cardiac function of rats with MI and CD73^+^AD-MSCs transplantation group appeared to be more effective (Figure 5 and Supplementary Figure 2).

### Effect of CD73^+^ AD-MSCs Transplantation on Angiogenesis

**FIGURE 6:**
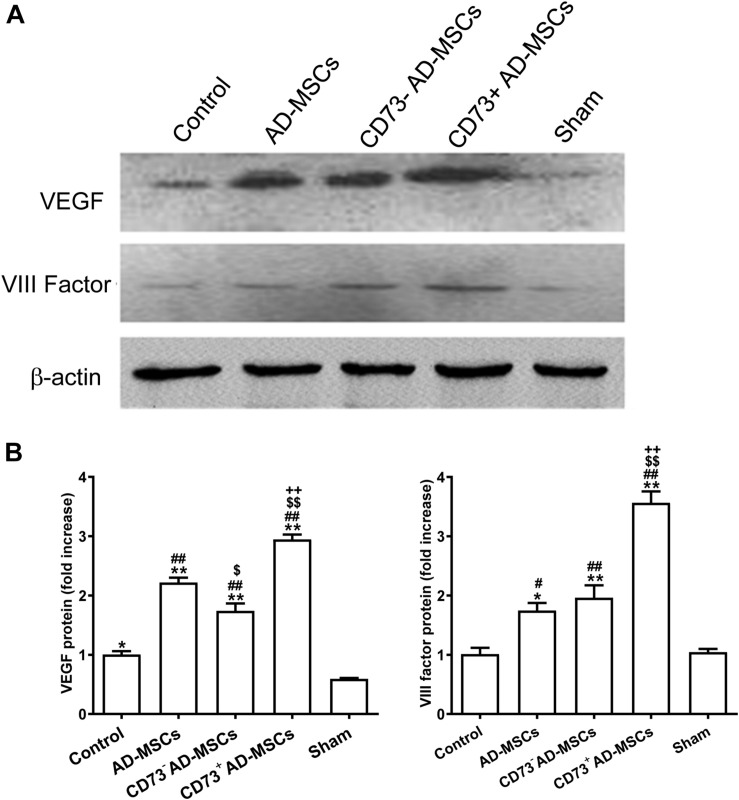
VIII factor and VEGF expression in myocardial infarction area after transplantation of AD-MSCs 4 weeks post MI. **(A)** Representative Western blots showing the level of VIII factor and VEGF under different treatments. **(B)** Quantification of the level of VIII factor and VEGF. *P < 0.05, **P < 0.01, vs. Sham; ^#^*P* < 0.05, ^##^*P* < 0.01, vs. Control; ^$^*P* < 0.05, ^$$^*P* < 0.01, vs. AD-MSCs; ^++^*P* < 0.01, vs. CD73^–^ AD-MSCs, *n* = 5∼6/group. One-way ANOVA, then followed by post Tukey’s test for multiple comparisons. Data are presented as mean ± SD.

Immunohistochemical staining and western blotting was used to detect the expression of factor VIII and VEGF, which marked vascular endothelial cells. The higher expression of VEGF and factor VIII were detected in the myocardial infarction area in the cell transplantation group, and the highest expression was found in the CD73^+^AD-MSCs transplantation group (*P* < 0.05) (Figure 6). By the end of the 4th week post MI, the neovascularization density in all transplantation groups was significantly higher than in the untreated MI group (*P* < 0.01). However, the extent of increase in CD73^+^ AD-MSCs group was higher than other transplanting groups (*P* < 0.05) (Supplementary Figure 3). These results indicate that transplantation of CD73^+^ AD-MSCs were more effective in promoting myocardial angiogenesis.

## Discussion

**FIGURE 7:**
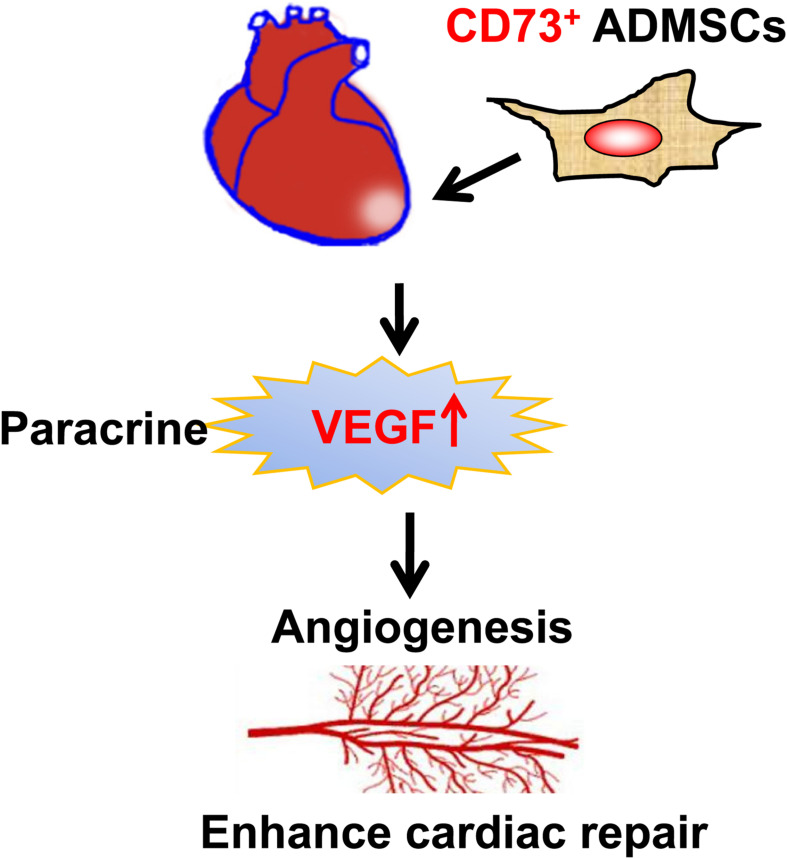
The diagram of the mechanisms responsible for improved cardiac repair after myocardial transplantation of CD73 positive AD-MSCs.

Unlike most similar studies, the present study utilized CD73^+^ and CD73^–^ subsets of AD-MSCs for transplantation. We demonstrated that the presence of CD73 promotes the secretion of VEGF, SDF-1α, and HGF factors by AD-MSCs *in vitro*. CD73^+^ AD-MSCs promoted vascular regeneration *in vivo*, which could be closely related to regulate the microenvironment of MI area. We also have showed that CD73^+^ AD-MSCs were more effective than bulk AD-MSCs in accelerating cardiac function recovery in the rat model of MI (Figure 7).

CD73 is a glycoprotein that acts as an exogenous -5′-nucleotide enzyme involved in signal transduction. CD73 hydrolyzes 5′-adenosine monophosphate (AMP) into adenosine and phosphoric acid and then interacts with adenosine receptors on the cell surface to modulate a variety of biological effects (Meng et al., 2019). CD73 also has non-hydrolase function, which is also a signal and adhesion molecule that regulates cell-extracellular matrix interaction (Yan A. et al., 2019). Moreover, anti-CD73 inhibitory antibody for cancer immunotherapy is already carried out in clinical trials (Minor et al., 2019). However, the role of CD73 in regeneration therapy of MSCs is rather limited.

Routinely, many laboratories tend to isolate and enrich MSCs through plastic adhesion when separating large numbers of MSCs (Li et al., 2016). In this study, CD73^+^ AD-MSCs and CD73^–^ AD-MSCs were separated by flow cytometry. The CD73^+^cells had either spindle or rod shape, while CD73^–^ cells mostly large and polygonal, in line with previous observations (Haasters et al., 2009). Although the transcriptome differences between MSCs from different sources are very small, they are sufficient to significantly affect cell behavior (Elahi et al., 2016). Gene chip analysis of this study revealed a number of differentially expressed genes in the subgroups. It showed differences between CD73^+^ AD-MSCs and CD73^–^AD-MSCs when the transcription profiles of these two subgroups were compared, especially in VEGF pathway. Therefore our data suggested that evaluation based on CD73, the surface marker, that can be used as a quality control identification standard for cell expansion *in vitro*.

Mesenchymal stem cells promote tissue repair by secreting soluble cytokines, promoting vascular regeneration, blocking apoptosis, suppressing inflammation, and stimulating the regeneration of host cells (Tsuji et al., 2010; Zhao et al., 2019). In this study, we transplanted cultured AD-MSCs into rats with MI and found that the purified CD73^+^ADMSCs significantly increased vascular density, promoted angiogenesis, improved ventricular remodeling and cardiac function when it was compared with mixed ADMSCs and CD73^–^ADMSCs. The therapeutic effect was good. Therefore the use of purified cell subsets could improve the pertinence of the study and the efficacy of the treatment.

Inhibition of CD73 with anti-CD73 antibody or APCP reduced tumor angiogenesis in mice (Allard et al., 2017). The mechanism was probably related to CD73’s interaction with adenosine receptor A2B (Hesse et al., 2017). This interaction will stimulate vascular smooth muscle cells, endothelial cells, immune cells and tumor cells to secrete VEGF. Our results showed CD73^+^AD-MSCs were superior to unsorted AD-MSCs and CD73^–^AD-MSCs in promoting myocardial angiogenesis. ELISA results showed that CD73 expression could stimulate the secretion of VEGF, SDF-1α, and HGF. The scratch experiment results showed that CD73^+^AD-MSCs cellular products significantly promoted HUVEC migration and scratch wound healing to support angiogenesis. It is speculated that CD73 may contribute to angiogenesis in infarcted regions by upregulating the secretion of VEGF and SDF-1α in AD-MSCs.

SDF-1 is a well-known chemokine. It played pivotal role in activation, mobilization, normalization, and retention of hematopoietic stem cells (Zhang et al., 2018). SDF-1 could induce VEGF secretion in cells. CXCR4 was the receptor of SDF-1. Then these actual roles effectively regulate the angiogenic activity and homing capacity of endothelial progenitor cells (Sordi et al., 2005; Lau and Wang, 2011). These cytokines not only promoted vascular regeneration, but also protected the myocardium from hypoxia (Lemcke et al., 2018). Our results indicated that CD73^+^ AD-MSCs, AD-MSCs and CD73^–^AD-MSCs could significantly improve the cardiac function of rats with MI, but that CD73^+^AD-MSCs transplantation appeared to be more effective. Exogenously expressed SDF-1α/CXCR4 played a role in recruiting cardiac stem cells (CSC) and exogenous VEGF could accelerate cardiac repair partly through SDF-1α/CXCR4 (Tang et al., 2011). MSCs promotes effective angiogenesis through overexpressing SDF-1. This effect can inhibit congestive heart failure caused by MI. *In vivo*, MSC-stimulated SDF-1α expression in infarcted hearts resulted in massive mobilization and homing of bone marrow stem cells and CSCS (Tian et al., 2019). High expression of HGF also has an obvious cardioprotective effect, it can inhibit cardiocyte apoptosis, promote vascular regeneration, and act effectively in the process of anti-ventricular remodeling and repair (Sonnenberg et al., 2015; Chang et al., 2016). Gallo et al. reported that the HGF/Met axis also acts effectively in adapting myocardial regeneration and self-renewal. This axis could enhance the number of cardiac progenitor cells (Gallo et al., 2015).

The analysis of the effects of MSCs transplantation in the treatment of myocardial injury was mainly focused on cytokine secretion and the differentiation ability of transplanted cells (Yan W. et al., 2019). Clearly, transplantation of AD-MSCs triggers multiple mechanisms that collectively improve heart function. Cytokines secreted from MSCs exert protective effects by salvaging injured neighboring cells through regulation of apoptosis, inflammation, fibrosis, and angiogenesis (Jung et al., 2017). In recent years, studies have shown that MSCs may be involved in the regulation of immune responses (Hara et al., 2019). CD73 and CD39 can be regarded as “immune checkpoint mediators” and can regulate the function of various types of immune cells (Allard et al., 2014). Our study showed that the expression of CD73 stimulated the production of HGF *in vitro*. In addition, HGF has obvious regulatory effects on endothelial cells, epithelial cells, and hematopoietic progenitor cells. it also has been shown to regulate the chemotaxis of T cells in heart tissue (Komarowska et al., 2015). Promoting angiogenesis and antiinflammation can interact, and VEGF expression is higher at low doses of TNF-α (Gomzikova et al., 2019). It is essential for myocardial repairing cell transplantation that CD73^+^ AD-MSCs regulate myocardial microenvironment especially by promoting angiogenesis.

## Conclusion

In the present study, our results demonstrated that CD73^+^ AD-MSCs revealed a higher pro-angiogenic paracrine activity and displayed therapeutic efficacy for MI therapy. These results suggested that CD73 could be used as a selection marker of MSCs for ischemic disease therapy. Further studies to investigate the intrinsic properties and therapeutic efficiency of CD73 and other subpopulations of MSCs will facilitate a new avenue for regenerative therapy. The strategy of using an MSC subpopulation offers a more effective and definitive methods for stem cell therapy.

## Data Availability Statement

The datasets presented in this study can be found in online repositories. The names of the repository/repositories and accession number(s) can be found below: NCBI GEO; GSE167219.

## Ethics Statement

The animal study was reviewed and approved by the ethical review approval number: XYLL-2015023, Ethics Committee of Xinxiang Medical University.

## Author Contributions

QL and ZL conceived, designed the experiment, and wrote the manuscript. ZG administered the project and provided financial support. HH, JG, ML, XY, and HZ collected and analyzed data. MZ edited the manuscript. All authors checked and approved the final manuscript.

## Conflict of Interest

The authors declare that the research was conducted in the absence of any commercial or financial relationships that could be construed as a potential conflict of interest.

## Supplementary Material

Supplementary Figure 1 Phenotypical characterizations of AD-MSCs using flow cytometer.

Supplementary Figure 2 Cardiac hemodynamic monitoring at weeks 1, 2, and 3. LVEF: Left ventricular ejection fraction; LVESV (ml): left ventricular end-systolic volume; LVEDV (ml): left ventricular end-diastolic volume. **P* < 0.05, ***P* < 0.01, vs. Sham; ^#^*P* < 0.05, ^##^*P* < 0.01, vs. Control; ^$^ *P* < 0.05, ^$$^*P* < 0.01, vs. AD-MSCs; ^+^*P* < 0.05, ^++^*P* < 0.01, vs. CD73^–^AD-MSCs, *n* = 5∼6. One-way ANOVA, then followed by post Tukey’s test for multiple comparisons. Data are presented as mean ± SD.

Supplementary Figure 3 VIII factor and VEGF expression in myocardial infarction area after transplantation of AD-MSCs 4 weeks post MI by IHC. **(A)** VEGF and **(B)** VIII factor staining revealed the promotion of angiogenesis by CD73 + AD-MSCs treatment. **P* < 0.05, vs. Sham; ^#^*P* < 0.05, vs. Control; ^[*d**o**l**l**a**r*]^*P* < 0.05, vs. AD-MSCs; ^+^*P* < 0.05, vs. CD73^–^ AD-MSCs, *n* = 5∼6. One-way ANOVA, then followed by post-Tukeypost-Tukeypost-Tukeypost-Tukeypost-Tukeypost-Tukeypost-Tukeypost-Tukey’s test for multiple comparisons. Data are presented as mean ± SD.

## Abbreviations

AD-MSCs: adipose derived mesenchymal stem cells
AMP: 5 ′ -adenosine monophosphate
APCP, alpha: beta-methyleneadenosine-5 ′ - diphosphate
bFGF: basic fibroblast growth factor
CXCR4: chemokine receptor-4
ECM: Extracellular matrix
FBS: Fetal bovine serum
HGF: hepatocyte growth factor
LAD: left anterior descending coronary artery
LVEDV: left ventricular end-diastolic volume
LVEF: left ventricular ejection fraction
LVESV: left ventricular end-systolic volume
MI: myocardial infarction
MSCs: mesenchymal stem cells
MOI: multiple of infection
SDF-1 α: stromal cell-derived factor-1 α
VEGF: vascular endothelial growth factor.
