# Supplementary material for: CD73+ Mesenchymal Stem Cells Ameliorate Myocardial Infarction by Promoting Angiogenesis
Source: Front Cell Dev Biol. 2021 May 12;9:637239. doi: 10.3389/fcell.2021.637239 (PMC8152667; doi:10.3389/fcell.2021.637239)
Supplement: Supplementary Figure 1 — Phenotypical characterizations of AD-MSCs using flow cytometer. [file Data_Sheet_1.docx]

**Supplemental information**

**
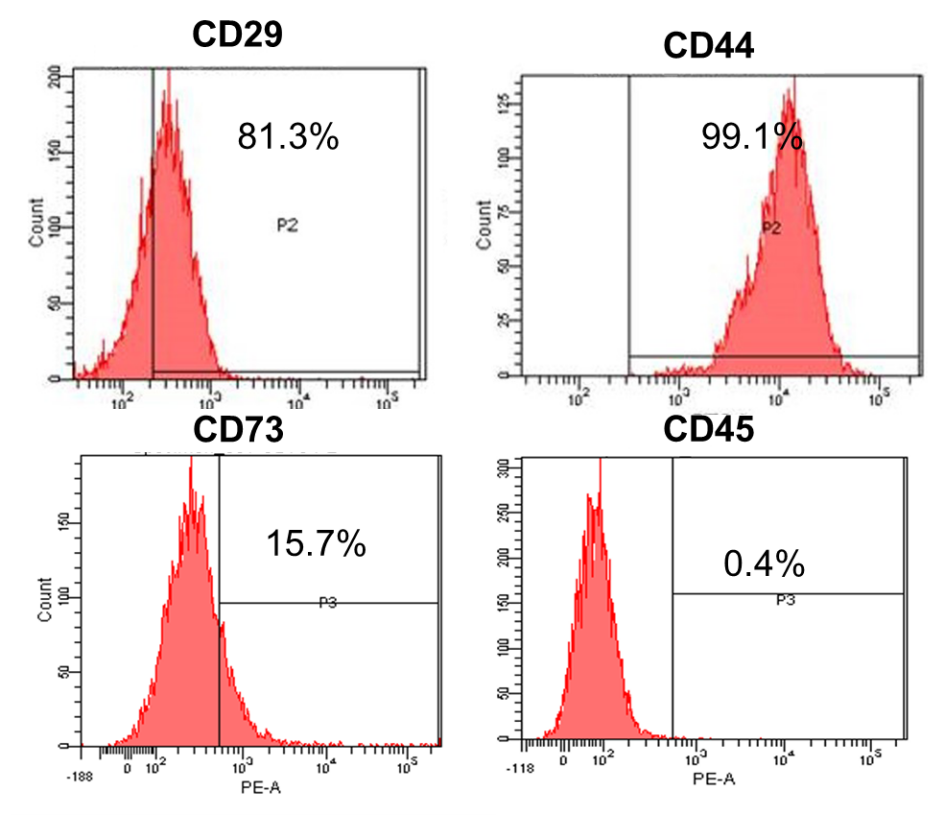
**

**Supplemental Figure 1. Phenotypical characterizations of AD-MSCs using flow cytometer.**

**
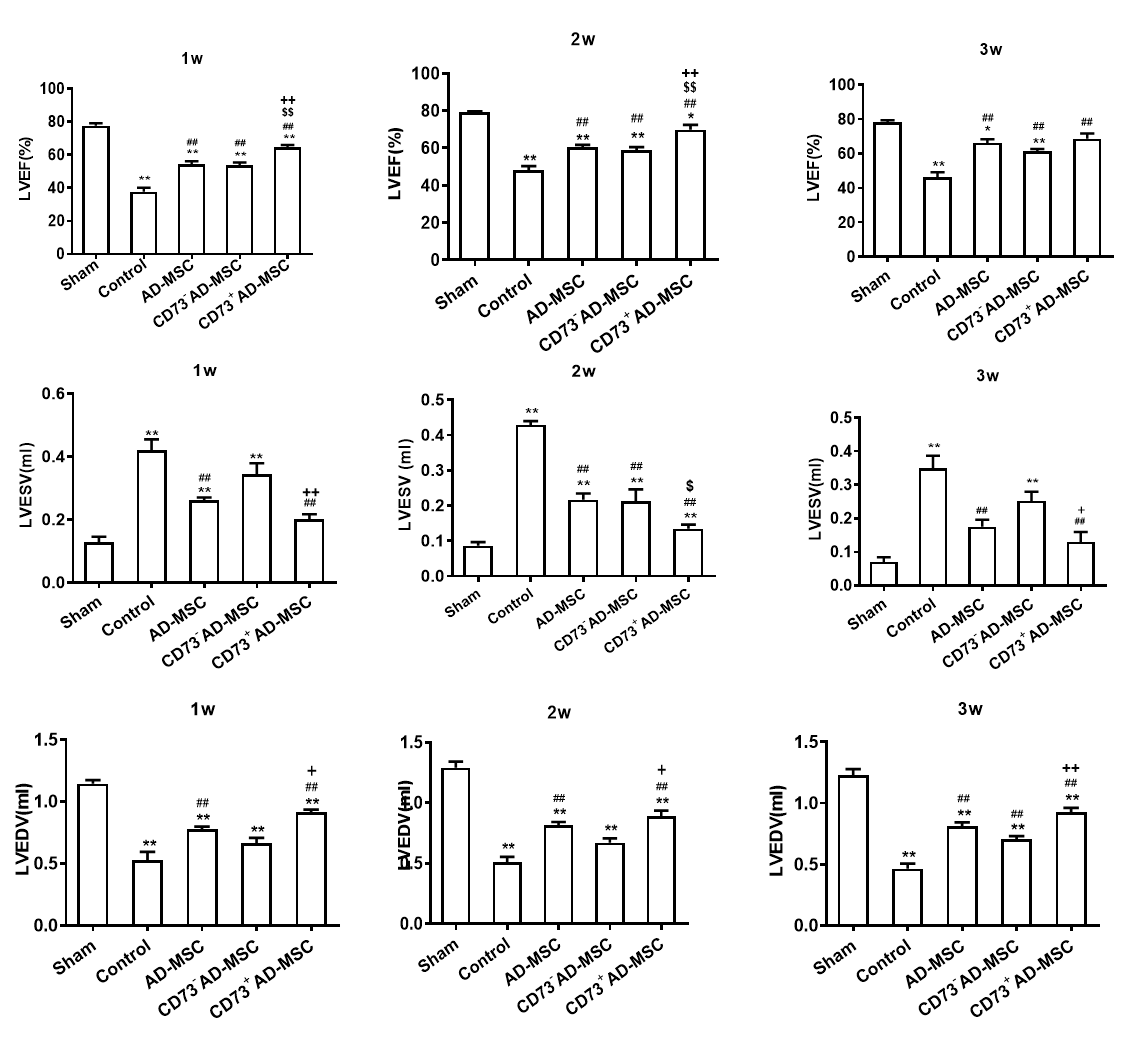
**

**Supplemental Figure 2. Cardiac hemodynamic monitoring at weeks 1, 2 and 3.** LVEF: Left ventricular ejection fraction; LVESV (ml): left ventricular end-systolic volume; LVEDV (ml): left ventricular end-diastolic volume. ^*^*P*<0.05, ^**^*P*<0.01, *vs.* Sham; ^＃^*P*<0.05, ^##^*P*<0.01, *vs.* Control (MI); ^$^ *P*<0.05, ^$$^*P*<0.01, *vs.* AD-MSCs (MI+AD-MSCs); ^+^*P*<0.05, ^++^*P*<0.01, *vs.* CD73^-^AD-MSCs (MI+ CD73^-^AD-MSCs), n=5~6. One-way ANOVA, then followed by post Tukey's test for multiple comparisons. Data are presented as mean ± SD.

**
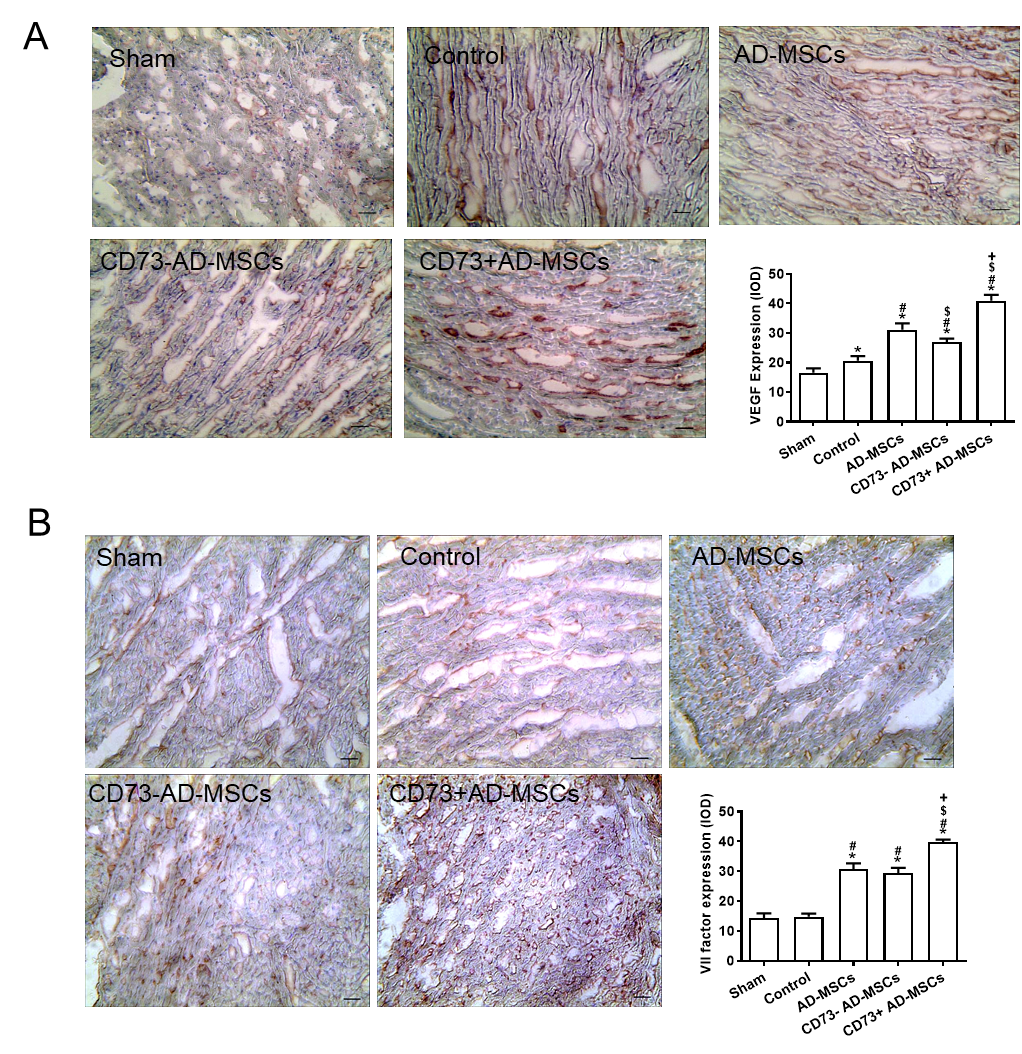
**

**Supplemental Figure 3.** VIII factor and VEGF expression in myocardial infarction area after transplantation of AD-MSCs 4 weeks post MI by IHC. (**A**) VEGF and (**B**) VIII factor staining revealed the effect of promoting angiogenesis by CD73+ AD-MSCs treatment. ^*^*P*<0.05, *vs.* Sham; ^＃^*P*<0.05, *vs.* Control (MI); ^$^ *P*<0.05, *vs.* AD-MSCs (MI+AD-MSCs); ^+^*P*<0.05, *vs.* CD73^-^ AD-MSCs (MI+CD73^-^AD-MSCs), n=5~6. One-way ANOVA, then followed by post Tukey's test for multiple comparisons. Data are presented as mean ± SD.
